# Female Genital Tuberculosis: Clinical Presentation, Current Diagnosis, and Treatment

**DOI:** 10.1155/2022/3548190

**Published:** 2022-11-18

**Authors:** Dian Tjahyadi, Bejo Ropii, Kevin Dominique Tjandraprawira, Ida Parwati, Tono Djuwantono, Wiryawan Permadi, Tinchiu Li

**Affiliations:** ^1^Department of Obstetrics & Gynaecology, Faculty of Medicine, Padjadjaran University, Bandung, Indonesia; ^2^Bandung Fertility Center, Limijati Women and Children Hospital, Bandung, Indonesia; ^3^Biomedical Engineering Research Group, School of Electrical Engineering and Informatics, Bandung Institute of Technology, Bandung, Indonesia; ^4^Department of Clinical Pathology, Faculty of Medicine, Padjadjaran University, Bandung, Indonesia; ^5^Assisted Reproductive Technology Unit Prince of Wales Hospital, Chinese University of Hong Kong, Central Ave, Hong Kong

## Abstract

Female genital tuberculosis is a disease caused by *Mycobacterium tuberculosis* infection in the female reproductive tract. The disease burden among women leads to infertility is significant, especially in developing countries. The bacteria can spread from the lung into the reproductive organ through lymphatic or hematogenous. Many patients present with atypical symptoms, which mimic other gynecological conditions. Several investigations are needed to establish the diagnosis. Almost all cases of genital TB affect the fallopian tube and cause infertility in patients and endometrial involvement. Current treatment still relies on antituberculosis therapy with a combination of tubal surgery. The present review describes the epidemiological data, clinical presentation, diagnosis, and currently available treatment to cure the disease and for in vitro fertilization.

## 1. Introduction

Tuberculosis (TB) is one of the world's major health problems. It affected about 10 million people in 2017 and caused approximately 1.5 million deaths [1]. Thirty countries with a high TB burden accounted for 87% of new TB cases. Two-thirds of the total cases were contributed by eight countries, with India leading the count, followed by Indonesia, China, the Philippines, Pakistan, Nigeria, Bangladesh, and South Africa. While 56 percent of TB cases and deaths occur among males, the disease burden is also high among females [2]. Female genital TB (FGTB) is an essential factor for infertility in countries with a high TB prevalence. This type of TB usually occurs secondary to primary pulmonary TB. The incidence of FGTB is increasing among young women globally. The spread is generally through hematogenous or lymphatic routes or direct spread. Tuberculosis infection in female genital organs can result in infertility. The therapy for FGTB is similar to the standard treatment used for pulmonary TB by giving antitubercular drugs. Early FGTB detection followed by suitable treatment with adequate dosages of antitubercular therapy can reduce damage and future infertility in these women. This article reviews the epidemiology, clinical presentations, diagnosis, and treatment of FGTB.

## 2. Epidemiology and Pathogenesis

Epidemiological and clinical data on FGTB cases are difficult to obtain. Challenges persist in the lack of clinical awareness of the possibility of FGTB, accurate diagnosis, and nonspecific clinical manifestations of the disease. Thus, the exact prevalence of FGTB in various geographical locations and specific patient groups is difficult to estimate because many patients remain asymptomatic and undiagnosed. Better indices are required to describe the epidemiological data of FGTB. These include indices for (a) new FGTB cases among patients admitted for gynecological disease and (b) FGTB cases among populations of infertile women. The incidence is very high in developing countries compared to developed countries. Extrapulmonary TB represented about 16% of the 7.1 million incident cases in 2019, ranging from 8% in the WHO Western Pacific region to 24% in the Eastern Mediterranean region [2]. Some study shows that the prevalence of FGTB varies, as seen in Table 1. The presence of asymptomatic FGTB cases may influence the reported estimates. Table 1 shows the literature on the prevalence of genital TB among women.

The route of *M. tuberculosis* infection of FGTB is usually through the hematogenous or lymphatic spread from pulmonary TB and possibly by direct spread from infected pelvic organs. Tuberculosis affects every part of the female reproductive tract. In India, the frequency of female genital organs affected by TB are fallopian tubes (95–100%); uterine endometrium (50–60%); ovaries (20–30%); cervix (5–15%); uterine myometrium (2.5%); and vagina and/or vulva (1%) [3]. A similar result was reported in a retrospectively collected case of FGTB diagnosed from 2006 to 2016 at the Department of Pathology of Hassan II University Hospital, Fes, Morocco. It showed that the most involved genital organs were fallopian tubes (63.84%), followed by ovaries (46.15%), endometrium (38.46%), and the cervix (23.07%) [4]. Both fallopian tubes were involved in most ( >90%) women in FGTB, and the involvement could be TB endosalpingitis, exosalpingitis, interstitial TB salpingitis, or salpingitis isthmic nodosa.

## 3. Clinical Presentation

The clinical presentation of FGTB depends upon the site of infection. About 40% of infertile women with FGTB are asymptomatic [5]. The most common presentation of FGTB is infertility due to blocked or damaged fallopian tubes, very low endometrium receptivity, or ovarian damage with low ovarian reserve and volume. Most patients have experienced chronic lower abdominal and pelvic pain accompanied by several characteristics such as pelvic mass, cyst, abscess, dyspareunia, menstrual dysfunction, dysmenorrhea, or postmenopausal bleeding. Finding these symptoms in female patients with infertility may lead to the FGTB.  Table 2 summarizes the signs and symptoms of FGTB from several studies.

## 4. Diagnosis

FGTB diagnosis requires confirmation from a combination of tests.  Table 3 shows that various methods are commonly used to diagnose FGTB. Gynecologists may start by gaining information through patient history, general physical examination, abdominal examination, and gynecological examination. Chest X-ray (CXR) may show active pulmonary or old healed lesions of past TB, leading to suspicions of genital involvement. Ultrasonography (USG) is also of immense diagnostic value. USG may show heterogeneous and thin endometrium, endometrial fluid, calcification, or bands and intrauterine synechiae in endometrial tuberculosis. USG can also demonstrate the presence of hydrosalpinges with cogwheel signs and inhomogeneous enlarged ovaries with free peritoneal fluid and fixed adnexal masses [17]. Hysteroscopy and laparoscopy are performed as indicated. Hysteroscopy is used to visualize the endometrial cavity and may look normal in the absence of endometrial TB. Usually, there is a pale-looking cavity, varying grades of the intrauterine, presence of tubercles, and small white caseous nodules. Since the fallopian tubes are involved in 90% of FGTB cases, laparoscopic findings may improve diagnosis. These findings include miliary tubercles; white, yellow, and opaque patches on the uterus, tubes, ovaries, and peritoneum; small swollen tubes with agglutinated fimbriae; tubal block; hydrosalpinx; tube dilatation; a fusion of fimbria end; calcified tubes; and abdominal and pelvic adhesions.

A study of 85 women with genital TB who underwent diagnostic laparoscopy showed various findings on laparoscopies, such as tubercles on the peritoneum or ovary, tube-ovarian masses, caseous nodules, and encysted ascites. Various grades of pelvic adhesions were also observed. Some fallopian tubes might look normal, have tubercles, caseous granuloma, pyosalphinx, hydrosalpinx, beaded tube, or are unable to be visualized [18]. Hysteroscopy findings in four cases showed a caseation, fibrotic and synechia, and thin endometrium due to the destruction of the endometrium caused by chronic inflammation of TB. The morphological features of inflammation in the endometrium are hard to be found because the endometrium is shed every month due to menstruation. Inadequate granuloma formation and acid-resistant bacilli on the Ziehl–Neelsen examination might be rarely found. Therefore, specimens should be collected from multiple sites of the endometrium [19]. Hysteroscopic observations in 348 consecutive cases of female genital tuberculosis showed some normal findings, pale endometrial cavity, TB sequelae like obstructed ostia, bilateral or unilateral periosteal fibrosis, endometrial glands atrophy, small shrunken cavity, distorted cavity, and various grades of intrauterine adhesions [20].

These tests could be combined with biomolecular assays such as Mantoux/tuberculin, interferon-gamma release assay (IGRA), and microbial test from the specimen to confirm the presence of TB. The microbial test may be performed using standard polymerase chain reaction (PCR) or GeneXpert to detect *M. tuberculosis* nucleic acid, bacterial culture, or enzyme-linked immunosorbent assay (ELISA) of mycobacterial antibodies. The combination of tests will improve diagnostic accuracy, but this ultimately depends on the quality of the samples. A composite reference standard might be used to make a final diagnosis based on the results of two or more tests. The Central TB Division and Directorate of Health Services, Ministry of Health and Family Welfare, Government of India has also developed a guideline to suspect, diagnose, and manage extrapulmonary TB [21].

## 5. Treatment

Multiple drug therapy in adequate doses and sufficient duration is the main treatment for FGTB. Short-course combination therapy for 6–9 months is an effective medical treatment for FGTB [22]. A randomized controlled trial on 175 women with FGTB showed no difference between six months versus nine months of antituberculous therapy in complete cure rate, recurrent rate, and pregnancy rate [23]. For new cases, whether microbiologically confirmed or clinically diagnosed and drug-sensitive, previously treated patients, including nonresponders, failures, recurrent TB, and loss to follow up for one month after receiving one month of antitubercular therapy (ATT), are given combination therapy. Medical therapy with antitubercular drugs is effective for FGTB [24–29].

For patients undergoing IVF, additional surgery might be required to improve the chances of conception. A 31-year-old woman with FGTB and fallopian tube involvement underwent a laparoscopic salpingostomy followed by regular ATT for two years. In vitro fertilization-embryo transfer (IVF-ET) gave birth to a healthy baby girl through vaginal delivery after 36 weeks of pregnancy [24]. A comparative study of 38 infertile women with FGTB undergoing salpingectomies and treated with ATT for 6-12 months demonstrated that salpingectomy is a definitive and effective treatment option. The surgery improved patients' clinical pregnancy and take-home baby rates after receiving ATT for 12 months [25].

The presence of hydrosalpinges may have a direct effect, such as embryotoxicity, lower endometrial receptivity, and the possible flush of the embryo from the uterus due to tubal fluid. A meta-analysis study showed that salpingectomy improves clinical pregnancy significantly compared to no treatment [30]. The clinical pregnancy was increased with salpingectomy for hydrosalpinges prior to IVF [31]. Another study showed that laparoscopic tubal occlusion could be used as an alternative to laparoscopic salpingectomy in improving IVF pregnancy rates in women with hydrosalpinges [32].

Fertility following FGTB remains a prickly issue. A study of 155 women with genital tuberculosis consisting of 25 patients with endometrial tuberculosis and 130 patients with tubal tuberculosis were treated with ATT used for pulmonary tuberculosis. The patients received a combination of isoniazid, rifampicin, ethambutol, and pyrazinamide for 2 months, followed by isoniazid and rifampicin for the next 4–10 months. The patients then underwent IVF or intracytoplasmic sperm injection (ICSI). Notably, patients with endometrial tuberculosis showed significantly reduced fertilization, implantation, and cumulative pregnancy rates, probably due to relatively lower endometrial thickness, embryo quality, and implantation rate (*p* < 0.05). However, the study failed to show any significant difference in pregnancy outcomes between endometrial and tubal tuberculosis patients. Additional studies are required to assess the structural and physiological damage in the endometrium caused by TB infection. Additional treatments may also be required to improve endometrial receptivity. Currently, ICSI-ET remains the most optimal method for treating female infertility associated with tubal tuberculosis [26].

Before performing IVF in infertile patients, it is essential to rule out the presence of FGTB to avoid TB dissemination during pregnancy. FGTB might develop during pregnancy and impair IVF outcomes. Assisted reproductive technology allows the possibility of congenital TB if the mothers are not properly evaluated and treated before the implantation of the embryos. Genital TB should be considered in infertile women in endemic countries, and screening for TB should be extensively done before the IVF procedure [33]. Early ATT in FGTB improved menstrual cycle, endometrial thickness, and reduced incidence of grade I adhesions [34]. In addition, salpingectomy or tubal clipping may be considered to improve pregnancy outcomes.

## 6. Conclusion

The prevalence of FGTB disease among infertile women is unknown yet likely to be of a significant burden. FGTB causes infertility and gynecological symptoms such as menstrual dysfunction and chronic pelvic pain. Diagnosis is made through a thorough history, clinical and gynecological examination, and various methods to detect the presence of *M. tuberculosis* infection. ATT in adequate doses of sufficient duration is the primary treatment for FGTB. For patients who will perform IVF, tubal surgery may be considered to avoid ectopic pregnancy and improve both clinical pregnancy and take-home baby rates in patients who have received ATT.

## Figures and Tables

**Table 1:** Characteristics of quoted studies.

Study design	Population	Num. of participants	FGTB prevalence	Infertility due to FGTB		Ref.	Authors
				Primary	Secondary		
Observational analytical study	Infertile woman	150	20%	83.33%	16.67%	[5]	(Shahzad, 2012)
Cross-sectional survey	Fertile and infertile woman	2778	0.9%	N/A	N/A	[6]	(Ali and Abdallah, 2012)
Community-based cross-sectional survey	Fertile and infertile woman	460	0.045%	N/A	N/A	[7]	(Parvez et al., 2017)
A descriptive, cross-sectional study	Infertile woman	193	6.7%	84.6%	15.4%	[8]	(Zahoor et al., 2019)
Prospective study	Infertile woman	200	5.5%	N/A	N/A	[9]	(D. Sharma et al., 2019)
Prospective study	Infertile woman	150	7.2%	N/A	N/A	[10]	(Jindal, 2006)
Prospective study	General gynecological problems	47	2.8%	N/A	N/A	[10]	(Jindal, 2006)
Meta-analysis	Infertile women	4361	24.2%	N/A	N/A	[11]	(Chaman-Ara et al., 2016)
Meta-analysis	General population of Iran	11756	6.3%	N/A	N/A	[12]	(Akbarizade et al., 2018)
A cross-sectional survey	Women presenting with various gynecologic symptoms	2778	1.6%	N/A	N/A	[6]	(Ali and Abdallah, 2012)
Prospective study	Tuberculosis patients	1672	1.25%	N/A	N/A	[13]	(Patel and Dhand, 2016)

**Table 2:** Symptoms and signs in FGTB.

Symptoms	Ref.	Authors
Lower abdominal pain	[5, 6, 10]	(Shahzad, 2012), (Ali and Abdallah, 2012), and (Jindal, 2006)
Menstrual disorders	[5, 6]	(Shahzad, 2012) and (Ali and Abdallah, 2012)
Leukorrhea (vaginal discharge)	[5, 7, 10, 14]	(Shahzad, 2012), (Parvez et al., 2017), (Jindal, 2006), and (Djuwantono et al., 2017)
Weight disturbances	[5]	(Shahzad, 2012)
Low-grade fever	[5]	(Shahzad, 2012)
Pelvic mass	[5–7]	(Shahzad, 2012), (Ali and Abdallah, 2012), and (Parvez et al., 2017)
Ruptured ectopic pregnancy	[5]	(Shahzad, 2012)
Chronic pelvic pain	[6, 7, 10, 14]	(Ali and Abdallah, 2012), (Parvez et al., 2017), (Jindal, 2006), and (Djuwantono et al., 2017)
Dysmenorrhea	[6, 10, 14]	(Ali and Abdallah, 2012), (Jindal, 2006), and (Djuwantono et al., 2017)
Postmenopausal bleeding	[6, 14]	(Ali and Abdallah, 2012), (Djuwantono et al., 2017)
Menorrhagia	[7]	(Parvez et al., 2017)
Infertility	[5–7, 10, 14]	(Shahzad, 2012),(Ali and Abdallah, 2012),(Jindal, 2006), (Parvez et al., 2017), and (Djuwantono et al., 2017)
Oligomenorrhoea	[7]	(Parvez et al., 2017)
Pyrexia	[10, 14]	(Jindal, 2006) and (Djuwantono et al., 2017)
Irregular and hard endometrium	[4]	(Efared et al., 2019)
Distended fallopian tube, thick wall	[4]	(Efared et al., 2019)

**Table 3:** Test combination for the diagnosis of genital tuberculosis.

Diagnostic aid	Ref.	Authors
Gynecologic symptoms	[5–10, 13, 14]	(Shahzad, 2012), (Ali and Abdallah, 2012), (Parvez et al., 2017), (Zahoor et al., 2019), (D. Sharma et al., 2019), (Jindal, 2006), (Patel and Dhand, 2016), and (Djuwantono et al., 2017)
History of tuberculosis	[5, 6, 10, 14]	(Shahzad, 2012), (Ali and Abdallah, 2012), (Jindal, 2006), and (Djuwantono et al., 2017)
Family history of tuberculosis	[5, 6, 10]	(Shahzad, 2012), (Ali and Abdallah, 2012), and (Jindal, 2006)
ESR	[5]	(Shahzad, 2012)
Chest X-ray showing evidence of past or present tuberculosis	[5, 6, 9, 10, 13]	(Shahzad, 2012), (Ali and Abdallah, 2012), (D. Sharma et al., 2019), (Jindal, 2006), and (Patel and Dhand, 2016)
Mantoux test	[5, 9, 10, 13]	(Shahzad, 2012), (D. Sharma et al., 2019), (Jindal, 2006), and (Patel and Dhand, 2016)
Ultrasound scan	[5, 9, 13]	(Shahzad, 2012), (D. Sharma et al., 2019), and (Patel and Dhand, 2016)
Histopathology	[5–10, 13]	(Shahzad, 2012), (Ali and Abdallah, 2012), (Parvez et al., 2017), (Zahoor et al., 2019), (D. Sharma et al., 2019), (Jindal, 2006), and (Patel and Dhand, 2016)
Ultrasonography	[8, 13, 14]	(Zahoor et al., 2019), (Patel and Dhand, 2016), and (Djuwantono et al., 2017)
AFB smear	[7–10, 13]	(Parvez et al., 2017), (Zahoor et al., 2019), (D. Sharma et al., 2019), (Jindal, 2006), and (Patel and Dhand, 2016)
Culture	[6–8, 13]	(Ali and Abdallah, 2012), (Parvez et al., 2017), (Zahoor et al., 2019), and (Patel and Dhand, 2016)
GeneXpert	[8, 9, 15]	(Zahoor et al., 2019), (D. Sharma et al., 2019), and (J. Sharma et al., 2020)
PCR	[7, 9]	(Parvez et al., 2017) and (D. Sharma et al., 2019)
Hysterosalpingography	[10, 13]	(Jindal, 2006) and (Patel and Dhand, 2016)
ELISA IgG and IgM against A60 mycobacterial antigen	[10]	(Jindal, 2006)
Laparoscopy	[13, 16]	(Patel and Dhand, 2016) and (Malhotra et al., 2020)

## Data Availability

The data supporting this review are from previously reported studies and datasets, which have been cited.
